# Identification of a distinct association fiber tract “IPS-FG” to connect the intraparietal sulcus areas and fusiform gyrus by white matter dissection and tractography

**DOI:** 10.1038/s41598-020-72471-z

**Published:** 2020-09-23

**Authors:** Tatsuya Jitsuishi, Atsushi Yamaguchi

**Affiliations:** Department of Functional Anatomy, Graduate School of Medicine, Chiba University, 1-8-1 Inohana, Chuo-ku, Chiba, 260-8670 Japan

**Keywords:** Neuroscience, Anatomy

## Abstract

The intraparietal sulcus (IPS) in the posterior parietal cortex (PPC) is well-known as an interface for sensorimotor integration in visually guided actions. However, our understanding of the human neural network between the IPS and the cortical visual areas has been devoid of anatomical specificity. We here identified a distinctive association fiber tract “IPS-FG” to connect the IPS areas and the fusiform gyrus (FG), a high-level visual region, by white matter dissection and tractography. The major fiber bundles of this tract appeared to arise from the medial bank of IPS, in the superior parietal lobule (SPL), and project to the FG on the ventral temporal cortex (VTC) in post-mortem brains. This tract courses vertically at the temporo-parieto-occipital (TPO) junction where several fiber tracts intersect to connect the dorsal-to-ventral cortical regions, including the vertical occipital fasciculus (VOF). We then analyzed the structural connectivity of this tract with diffusion-MRI (magnetic resonance imaging) tractography. The quantitative tractography analysis revealed the major streamlines of IPS-FG interconnect the posterior IPS areas (e.g., IP1, IPS1) with FG (e.g., TF, FFC, VVC, PHA2, PIT) on the Human Connectome Project multimodal parcellation atlas (HCP MMP 1.0). Since the fronto-parietal network, including the posterior IPS areas, is recruited by multiple cognitive demands, the IPS-FG could play a role in the visuomotor integration as well as the top-down modulation of various cognitive functions reciprocally.

## Introduction

Vision-guided actions (e.g., reaching, grasping, and object manipulation) depend on the visual information to finely coordinate motor responses of the eye and hand to perform accurate, rapid, and tuned movements^1,2^. Since the lesions to the intraparietal sulcus (IPS) and the superior parietal lobule (SPL) in the posterior parietal cortex (PPC) result in the neurological deficit called Balint’s syndrome (optic ataxia)^2–4^, the IPS is considered as a hub for sensorimotor integration in visually guided actions, including eye–hand coordination. The IPS is also implicated in a wide range of cognitive and sensorimotor processes, including attention, working memory, and decision-making^5^. On the other hand, the fusiform gyrus (FG), on the ventral temporal cortex (VTC), covers a core network conducting different cognitive tasks (e.g., object recognition, visual language perception, visual attention) with lateralized specific object categories (e.g., words, faces, places, and bodies)^6,7^. However, our understanding of the neural network between the IPS areas and the visual cortex has been devoid of anatomical specificity.

In the present study, we identified a distinct association fiber tract described as “IPS-FG” that connects the IPS areas and the FG in the post-mortem brain dissection in four hemispheres (two right and left sides, respectively). This tract interconnects the IPS areas with the FG vertically in parallel between the arcuate fasciculus (AF) and the vertical occipital fasciculus (VOF). It courses in the temporo-parieto-occipital (TPO) junction, where several fiber tracts intersect in a complex way, including the superior longitudinal fascicle (SLF), the arcuate fasciculus (AF), the middle longitudinal fasciculus (MdLF), the inferior longitudinal fasciculus (ILF), the inferior fronto-occipital fasciculus (IFOF), and the VOF^8,9^. Bullock et al. recently identified four fiber tracts connecting dorsal-to-ventral cortical regions in the TPO junction by diffusion-MRI tractography; (1) VOF, (2) MdLF, (3) posterior AF (pAF), and (4) temporo-parietal connection to superior parietal lobule (TP-SPL)^10^. Our fiber dissection and tractography study showed the anatomical relationships among IPS-FG, AF, and VOF. In addition, we showed the IPS-FG overlaps with the border of the TP-SPL by tractography^9–11^.

## Results

### Identification of a distinct fiber tract “IPS-FG” to connect IPS areas and FG

**Figure 1:**
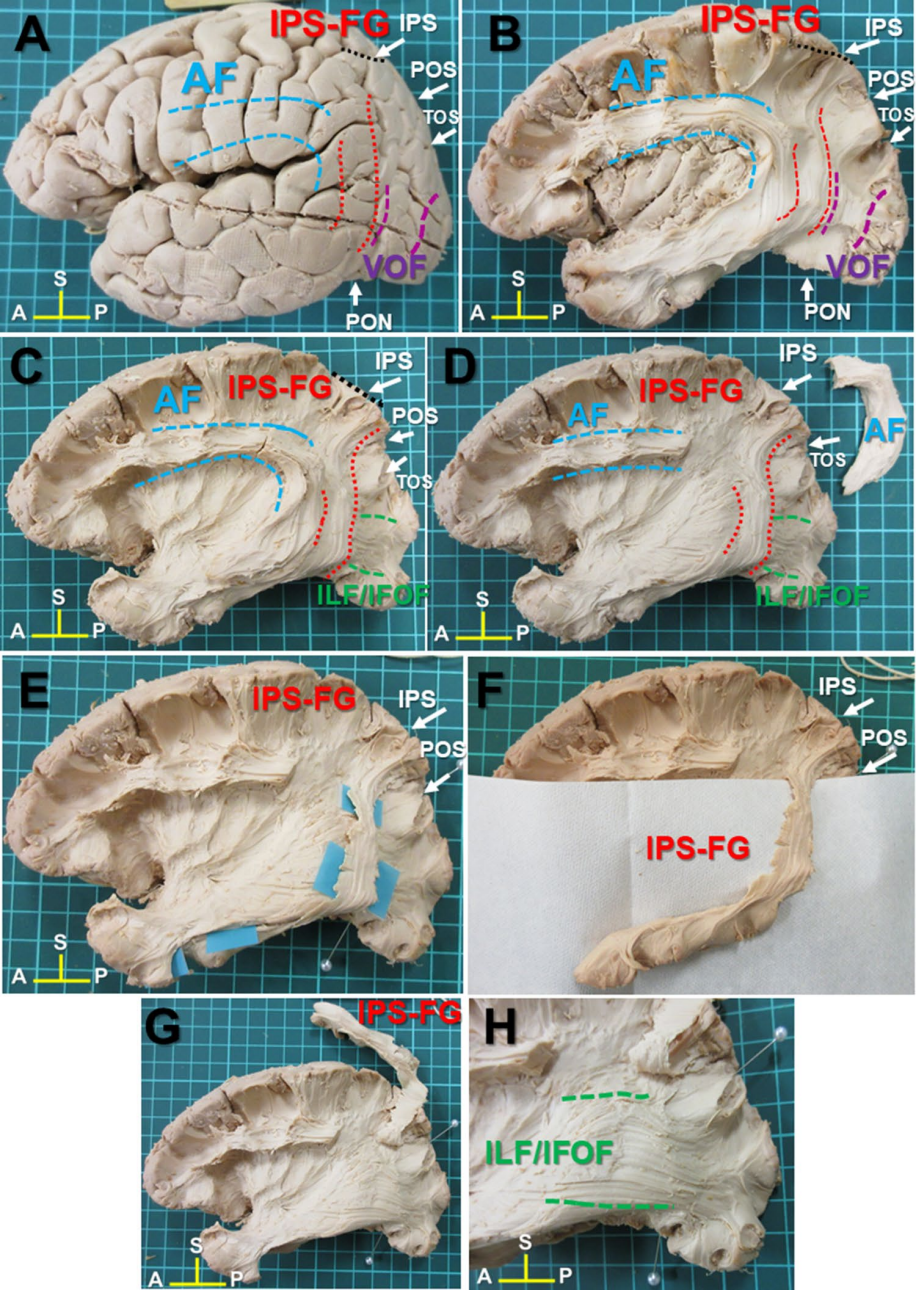
Lateral view of IPS-FG in the left hemisphere. (**A**) The lateral view of left hemisphere after removal of the meninges and vessels, with representative anatomical landmarks. (**B**) The lateral view after dissection to expose the fiber bundles of IPS-FG. The frontoparietal and temporal opercula around the insula were removed to expose AF and VOF. (**C**,**D**) Further dissection after removal of VOF (**C**) and AF (**D**). (**E**–**G**) Isolation of IPS-FG in the left hemisphere. (**H**) Magnified image of the lateral occipital cortex in the left hemisphere. *AF* arcuate fasciculus, *VOF* vertical occipital fasciculus, *ILF* inferior longitudinal fasciculus, *IFOF* inferior fronto-occipital fasciculus, *IPS* intraparietal sulcus, *POS* parieto-occipital sulcus, *TOS* transverse occipital sulcus, *PON* pre-occipital notch, *A* anterior, *P* posterior, *S* superior.

We recently reported the VOF could connect the dorsal and ventral visual stream by human white matter dissection^12^. The VOF is the lateral association fiber tract running vertically in the posterior-lateral corner of the brain^12,13^. During the dissection of VOF, we happened to identify a distinct fiber tract described as “IPS-FG”, which appeared to connect the areas around the IPS (IPS areas) and the ventral temporal cortex (VTC) (Fig. 1A,B). This fiber tract courses in parallel between the posterior portion of AF and the VOF (Fig. 1B), laterally to the ILF/IFOF in the occipital lobe (Fig. 1C). Since the fiber bundles of ILF and IFOF could not be differentiated each other by this superficial dissection, we labeled them as ILF/IFOF (Fig. 1C,D,H). To further delineate the trajectory of IPS-FG, we isolated the fiber bundles of AF which partially crossed over the IPS-FG. After removal of the AF, the whole image of IPS-FG’s trajectory became visible just anterior to the VOF (Fig. 1D). The major fiber bundles of the IPS-FG appeared to arise from medial border of the IPS on the SPL (superior parietal lobule) and project to the VTC through the temporo-parieto-occipital (TPO) junction (Fig. 1C,D). To further confirm the IPS-FG as a distinct association fiber tract, we isolated the fiber bundles from the cortex (Fig. 1E–G). After isolation of the IPS-FG, we could observe the fiber bundles of ILF/IFOF streaming antero-posteriorly beneath the IPS-FG (Fig. 1H). To replicate the results, we performed dissection in additional three brain hemispheres (two right sides and one left side) and could identify the fiber bundles of IPS-FG in all of them (Supplementary Figs. S1–S3).

### Anatomical relationships among IPS-FG, AF, and VOF

**Figure 2:**
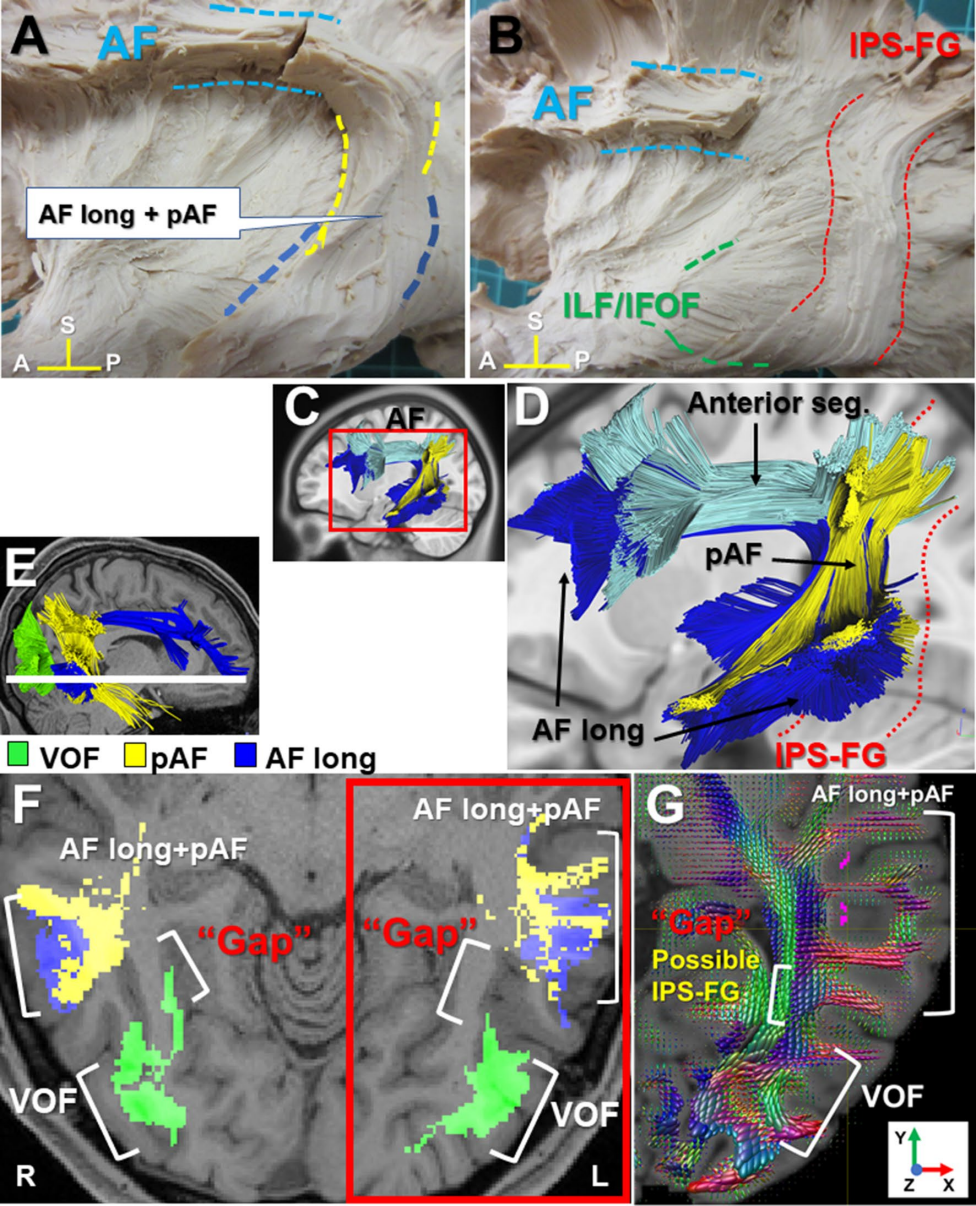
Anatomical relationships among IPS-FG, AF, and VOF at the TPO junction. (**A**,**B**) Magnified images of the insula and temporo-parieto-occipital (TPO) junction to show the anatomical relationships among IPS-FG, AF and ILF/IFOF. (**C**,**D**) Tractography of the perisylvian language networks in the left hemisphere in the brain template (HCP1021). They consist of three subcomponents (long segment [AF long], anterior segment, and posterior segment [pAF]). (**E**) Lateral view shows the tractography of pAF (yellow), AF long (blue), and VOF (green). The white line shows the level of axial image in (**F**). (**F**) The axial view indicates the anatomical relationships among pAF (yellow), AF long (blue), and VOF (green) tractography in the brain template (HCP1021). (**G**) Axial view of the fiber orientation distribution functions (colored by orientation) overlaid on the T1-weighted image in the temporo-occipital lobe, in which the blue color indicate the voxels with vertically oriented fascicles. This area corresponds to the red rectangle region in (**F**). *AF* arcuate fasciculus, *pAF* posterior AF, *VOF* vertical occipital fasciculus, *ILF* inferior longitudinal fasciculus, *IFOF* inferior fronto-occipital fasciculus, *A* anterior, *P* posterior, *S* superior.

The trajectory of AF courses in the TPO junction where seven fiber tracts intersect^8^. To investigate the anatomical relationship between IPS-FG and AF, we here showed the magnified dissection images of TPO junction (Fig. 2A,B). Catani et al. reported the perisylvian language networks consist of the direct (long segment [AF long]) and the indirect (anterior and posterior segment [pAF]) pathways by tractography, in which the indirect pathway runs parallel and lateral to the direct pathway^14^. We could observe the fiber tracts of perisylvian language networks in the tractography, corresponding to the direct pathway (AF long) and the indirect posterior segment (pAF) (Fig. 2C,D)^14,15^. In the dissection, the AF appeared to be a vertical sheet of fibers posterior to the Sylvian fissure, extending to the occipito-temporal lobe (Fig. 2A). To expose the IPS-FG, we removed the fiber bundles of possible “AF long” and “pAF” (Fig. 2B). The IPS-FG appeared to run just beneath and posterior to AF that covered over IPS-FG.

Figure 2E,F show the anatomical relationships among AF long, pAF, and VOF tractography in the sagittal and axial image of temporo-occipital lobe, respectively. Figure 2G represents the fiber orientation distribution functions (colored by orientation) for the rectangle region of Fig. 2F, in which the blue color indicates the voxels with vertically oriented fascicles. We found the “Gap” between VOF and AF in the axial image of tractography (Fig. 2F), which could correspond with the blue voxels (labeled as “possible IPS-FG”) between VOF and AF in Fig. 2G. These results indicate the IPS-FG could course vertically and medially between AF and VOF at the TPO junction.

### The cortical projections of IPS-FG on the dorsal and ventral cortex in dissection

**Figure 3:**
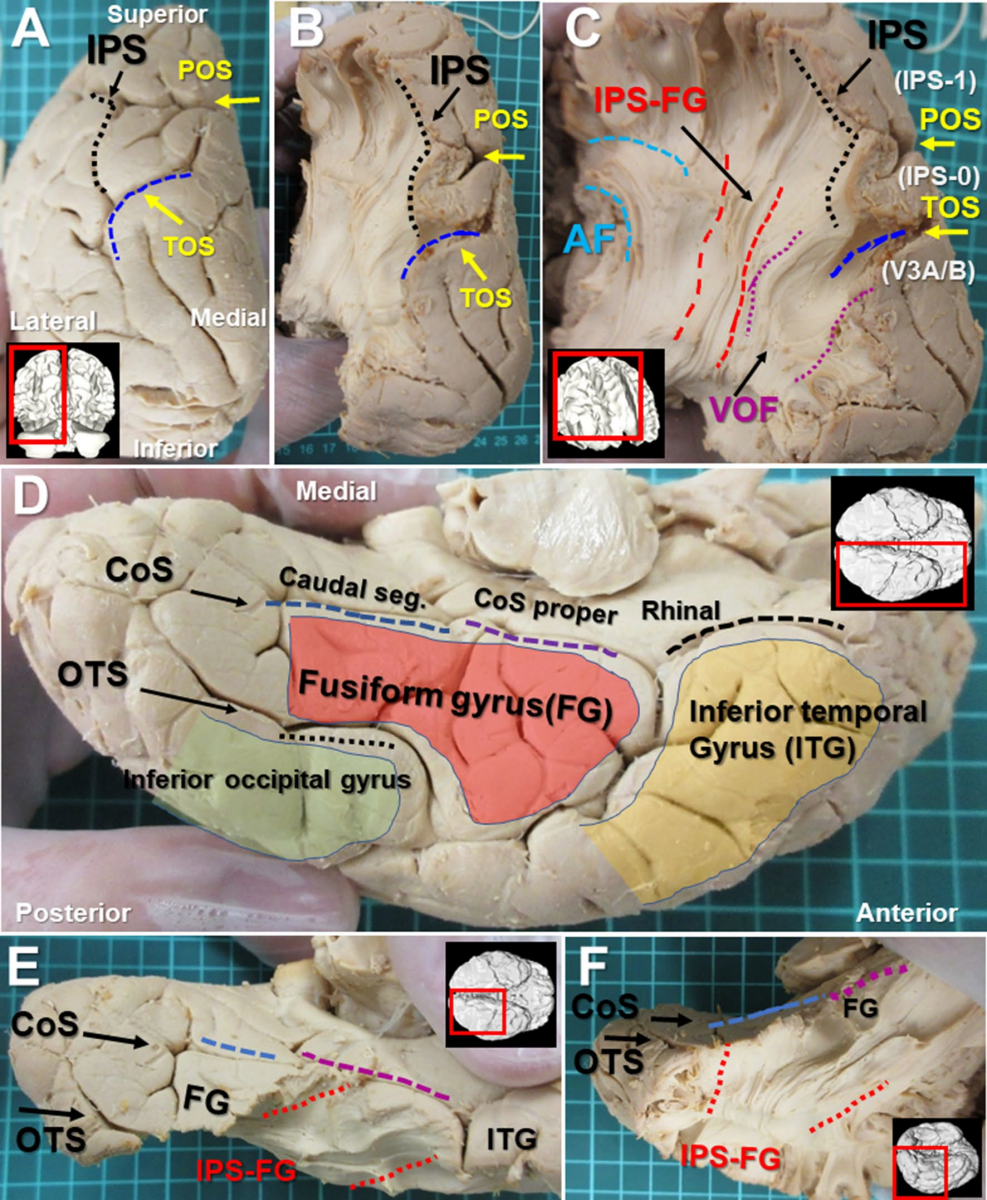
The cortical projections of IPS-FG on the dorsal and ventral cortex in dissection. (**A**,**B**) Caudal view of the left hemisphere after dissection of the cortex along the IPS and the TOS. (**C**) Posterolateral view of the brain after dissection to expose the fiber bundles of IPS-FG and VOF arising from IPS and TOS, respectively. “IPS-0, IPS-1, and V3A/B” indicate the anatomy-based visual filed maps. (**D**) Ventral temporal cortex (VTC) of the brain, showing the CoS (caudal, proper, and Rhinal segment), the OTS, the fusiform gyrus (orange), the inferior temporal gyrus (yellow), and the inferior occipital gyrus (green). (**E**,**F**) VTC of the brain after dissection of the cortex along the CoS to expose the fiber bundles of IPS-FG, running between CoS and OTS. *IPS* intraparietal sulcus, *TOS* transverse occipital sulcus, *POS* parieto-occipital sulcus, *CoS* collateral sulcus, *OTS* occipito-temporal sulcus, *FG* fusiform gyrus, *ITG* inferior temporal gyrus.

To investigate the dorsal cortical projection of this tract, we tracked down the fiber bundles toward the IPS (Fig. 3A–C). The major cortical projections of this tract, arising from the medial bank of IPS in the SPL (superior parietal lobule) (Fig. 3B), extended inferiorly in parallel with the VOF (Fig. 3C). The SPL, which the IPS borders medially, harbors at least five visual areas (IPS-0, IPS-1, IPS-2, IPS-3, IPS-4) by fMRI studies, starting from the most posterior IPS-0 that borders V3A/B, and extending along the medial bank of IPS^2,5^ (Fig. 3C). IPS-0 lies at the intersection of the parieto-occipital sulcus (POS) and IPS, adjacent to V3A/B areas (Fig. 3C). As we recently reported^12^, the cortical projections of VOF concentrated in the transverse occipital sulcus (TOS) at the bottom of the IPS (Fig. 3C), where the boundary of V3A/B areas are situated. Those of VOF also appeared to fall in the posterior IPS (i.e., IPS-0) (Fig. 3C). In contrast, the dorsal cortical projections of IPS-FG appeared to fall in anatomy-based IPS-1 anterior to IPS-0 area (Fig. 3C).

Next, to examine the ventral cortical projections, we tracked down the fiber bundles on the VTC (ventral temporal cortex) (Fig. 3D). The ventral cortical fiber bundles appeared to project broadly in the fusiform gyrus (FG) through the inferior occipital gyrus (Fig. 3D,E). The collateral sulcus (CoS) consists of three parts (the rhinal sulcus, CoS proper, and caudal segment)^16^, segregating the FG from the parahippocampal gyrus (Fig. 3D). The occipito-temporal sulcus (OTS) is a lateral border of the FG. The anterior portion of fiber bundles extended to the anterior border of FG, while the medial portion reached to the CoS (Fig. 3E,F). Thus, the anatomical anterior and medial border of this tract might be the anterior FG and the CoS, respectively.

To replicate these results, we performed dissection in additional three brain hemispheres (two right sides and one left side) (Supplementary Figs. S1–S3). In these brains, we could also observe the dorsal and ventral cortical projections fall in the medial bank of IPS (e.g., IPS-1) and the FG, respectively.

### Diffusion-MRI tractography of IPS-FG

**Figure 4:**
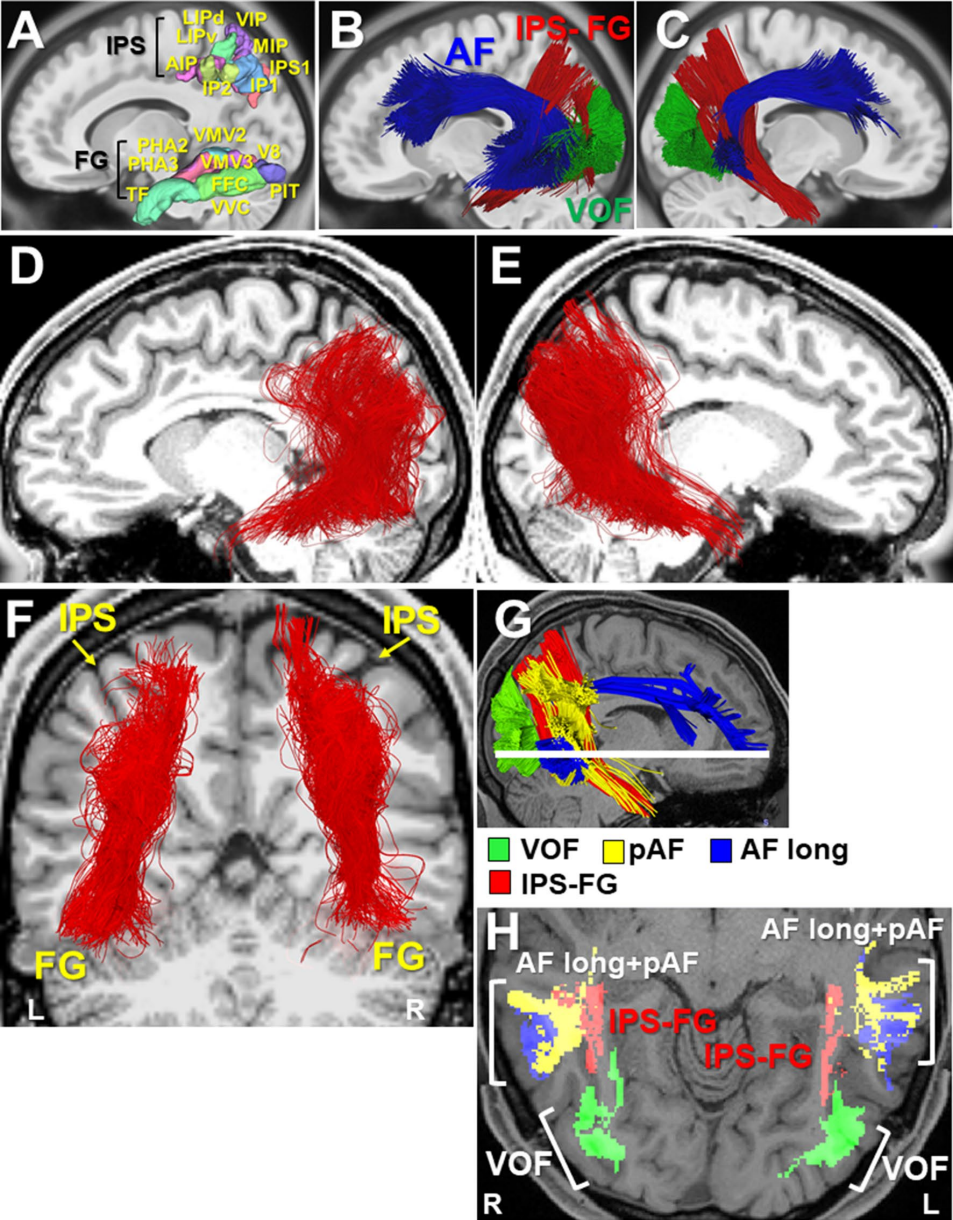
Reconstruction of IPS-FG tractography. (**A**) The ROIs of HCP MMP1.0 atlas, used for the fiber tracking of IPS-FG, were overlaid on the sagittal T1-weighted image. (**B**,**C**) Left and right lateral view of IPS-FG (red) with AF (blue) and VOF (green) tractography in the brain template (NTU-90), respectively. (**D**–**F**) Left and right lateral, and coronal view of the group-integrated tractography of IPS-FG overlaid on the sagittal T1-weighted image, respectively. (**G**) Lateral view of the IPS-FG (red), pAF (yellow), AF long (blue), and VOF (green) tractography in the brain template (HCP1021). The white line indicates the level of axial image in (**H**). (**H**) Axial view of tractography overlaid on the T1-weighted image to show the anatomical relationships among IPS-FG (red), pAF (yellow), AF long (blue), and VOF (green) in the temporo-occipital lobe. *AF* arcuate fasciculus, *pAF* posterior AF, *VOF* vertical occipital fasciculus, *IPS* intraparietal sulcus, *AIP* anterior intraparietal, *VIP* ventral intraparietal, *MIP* medial intraparietal, *IP1, 2* intraparietal 1, 2, *IPS1* intraparietal sulcus 1, *LIPv* lateral intraparietal ventral, *LIPd* lateral intraparietal dorsal, *PHA2, 3* parahippocampal area 2, 3, *V8* visual area 8, *PIT* posterior inferior temporal, *FFC* fusiform face complex, *VVC* ventral visual complex, *VMV2, 3* ventro-medial visual areas 2, 3.

To investigate the structural connectivity, we reconstructed the fiber tracts of IPS-FG by diffusion-MRI tractography. We used a tractography software tool (DSI studio) that utilizes a deterministic fiber tracking algorithm. We took a two-ROI approach to generate streamlines using publically available brain template, a mean data of 90 healthy subjects (NTU-90 brain atlas)^17^. The ROIs were selected from the cortical areas of Human Connectome Project multi-modal parcellation 1.0 (HCP MMP1.0), an updated human cortical map^18,19^. Using the IPS areas and the FG as two ROI masks (Fig. 4A), we could reconstruct the streamlines of IPS-FG running vertically and medially between AF and VOF in the brain template (Fig. 4B,C). We then generated the IPS-FG tractography using the data of 60 healthy subjects from HCP databank^18^. Figure 4D–F showed the lateral and coronal view of the group-level integrated tractography from 60 subjects’ data. To show the inter-individual variability, we presented the representative 5 subjects’ tractography in Supplementary Fig. S4. We confirmed the cortical projections of the IPS-FG tractography terminate in the IPS areas and the FG, respectively (Fig. 4F). To analyze the anatomical relationships among IPS-FG, AF, and VOF, we investigated the streamlines of each tract in the axial image of temporo-occipital lobe (Fig. 4G,H), which represent the same level images of Fig. 2E,F, respectively. Although there is some asymmetry between right and left hemisphere, the streamlines of IPS-FG (red) course vertically and medially between AF and VOF (Fig. 4G,H).

There are two broadly used algorithms of fiber tracking (i.e., deterministic and probabilistic tractography). The deterministic fiber tracking conducts the "maximum likelihood estimation" of the fiber tracks, while the probabilistic fiber tracking represents the possible distribution of the fiber tracts (https://dsi-studio.labsolver.org/). We utilizes the software tool DSI studio (deterministic algorithm) to generate the IPS-FG tractography. To compare the trajectory patterns between deterministic and probabilistic algorithm for cross-method validation, we then used the software tool MRtrix3.0 (probabilistic fiber tracking algorithm) for IPS-FG tractography in a representative subject^20^. Since the trajectory patterns of IPS-FG appeared similar between these two methods (Supplementary Fig. S5), we performed the following analyses by DSI studio.

### Quantitative tractography analysis of IPS-FG

**Figure 5:**
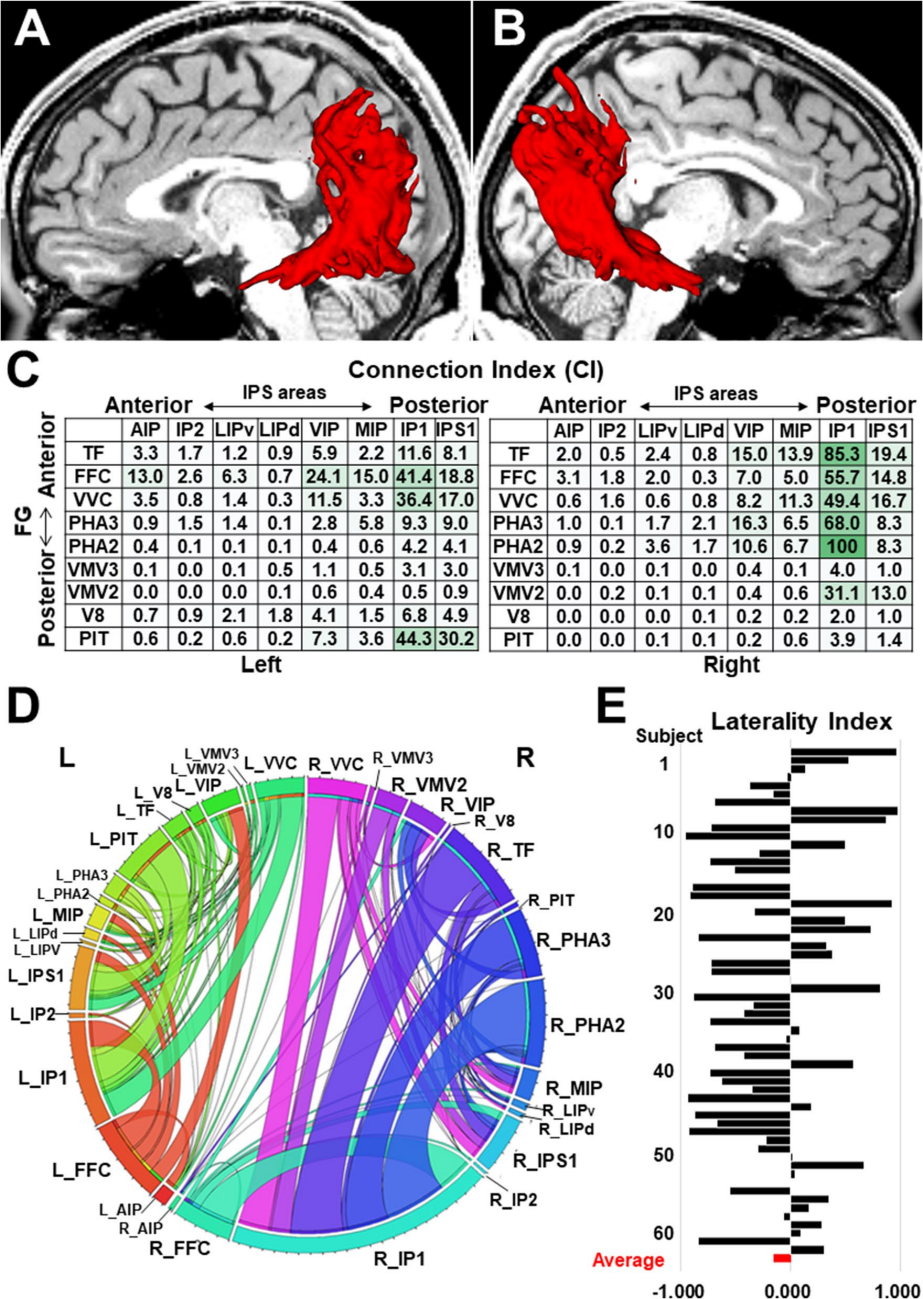
Streamline density maps, Connection index (CI), Connectogram, and Laterality index (LI). (**A**,**B**) Lateral view of streamline density maps for 60 subjects’ IPS-FG tractography, overlaid on T1-weighted image. (**C**) The tables indicate the Connection index (CI) for the tract numbers of each connection to link IPS areas and FG. Left; left hemisphere. Right; right hemisphere. Darker colors indicate the strength of connection. The data is average of healthy 60 subjects. (**D**) Connectogram representing bilateral connectivity patterns of IPS-FG, reflecting tract numbers of each connection. Circular color map represents the segmented brain regions that each streamline interconnects. The segmented arc and ribbon size represent the computed degrees of connectivity (the number of tracts) between segmented brain regions. The data is average of 60 subjects. (**E**) Graph indicates the laterality index (LI) for the tract numbers of IPS-FG in each subject (#1-60). The lowermost lane (red) is the average value of 60 subjects. The *laterality index* (left − right)/(left + right) shows the cerebral asymmetry. *AIP* anterior intraparietal, *VIP* ventral intraparietal, *MIP* medial intraparietal, *IP1, IP2* intraparietal 1, 2, *IPS1* intraparietal sulcus 1, *LIPv* lateral intraparietal ventral, *LIPd* lateral intraparietal dorsal, *PHA2, 3* parahippocampal area 2, 3, *V8* visual area 8, *PIT* posterior inferior temporal, *FFC* fusiform face complex, *VVC* ventral visual complex, *VMV2, 3* ventro-medial visual areas 2, 3.

To visualize the average spatial distribution of streamlines inside the IPS-FG, we here showed the streamline density maps of IPS-FG from 60 subjects of HCP data bank^18^ (Fig. 5A,B). The distributions of IPS-FG streamlines were observed to course vertically connecting the IPS areas and FG broadly, consistent with the group-level integrated tractography (Fig. 4D,E). To further gain insights into the structural connectivity, we analyzed the terminal projections of IPS-FG tractography in a subject-based approach using HCP datasets. Connection index (CI) is a value between 0 and 100, with 0 representing no connectivity, and 100 representing the strongest relative connectivity to a particular atlas region^21^. By calculating CI in the individual IPS-FG tractography, we assessed the relative strength to connect the IPS areas and FG. The average data of 60 subjects for each connection in the number and the volume were shown in Fig. 5C and Supplementary Fig. S6A, respectively. The stronger connections in the left hemisphere were those between the IPS areas (IP1, IPS1, VIP, MIP) and the FG (FFC, PIT, VVC, TF), while those in the right were connections between the IPS areas (IP1, IPS1, VIP, MIP) and the FG (TF, PHA2, PHA3, FFC, VVC) (Fig. 5C, Supplementary Fig. S6A).

To map and interpret the overview of IPS-FG’s structural connectivity, we employed a circular visualization method called ‘connectogram’, which constructs a circular representation of cortical networks^22^. We generated the connectograms to represent the connectivity patterns of streamlines in the number and in the volume using average data of 60 subjects, respectively (Fig. 5D, Supplementary Fig. S6B). Although there is slightly rightward asymmetry in some connections, the connectogram showed the bilateral stronger connections between the posterior IPS (i.e., IP1, IPS1) and the anterior-to-middle FG (i.e., TF, FFC, VVC, PHA2, PHA3). In addition, we observed the stronger connection between the posterior IPS (i.e., IP1, IPS1) and the posterior FG (i.e., PIT) specifically in the left hemisphere (Fig. 5C,D, Supplementary Fig. S6A,B).

To assess the laterality of IPS-FG tractography, we calculated the laterality index (LI) for the total number and volume of streamlines in 60 subjects, respectively. The laterality index (left − right)/(left + right) shows a cerebral asymmetry depending on the value. The positive values indicate the leftward, while the negative values show the rightward asymmetry. With the remarkable individual variability, the average LI was a small minus value (LI for the number of tracts, − 0.152; LI for the volume of tracts, − 0.037), indicating the slightly rightward asymmetry of IPS-FG (Fig. 5E, Supplementary Fig. S6C).

## Discussion

In the present study, we isolated a distinct association fiber tract “IPS-FG” by fiber dissection in four brain hemispheres, which vertically connects the IPS areas and the FG. We further analyzed the structural connectivity by tractography.

Anatomically, “IPS-FG” courses vertically and medially between AF and VOF at the TPO (temporo-parieto-occipital) junction, connecting the medial bank of IPS and the FG. Bullock et al. (2019) described the four fiber tracts to connect the dorsal-to-ventral cortical regions at the TPO junction by tractography, including VOF, MdLF, pAF, and TP-SPL (temporo-parietal connection to superior parietal lobule)^10^. Since the TP-SPL connects the SPL with the temporal lobe^9–11^, it could overlap with the fibers of IPS-FG. To assess this possibility, we investigated the anatomical relationship between TP-SPL and IPS-FG by tractography (Supplementary Fig. S7). The streamlines of IPS-FG appeared to course at the medial-inferior border of TP-SPL, merging in part with those of TP-SPL. In addition, previous studies showed the TP-SPL terminates near the fusiform gyrus^9–11^. These results support the IPS-FG could overlap with the border of TP-SPL. With quantitative T1 mapping, Schurr et al*.* described the vertical streamlines anterior to VOF project to more superior parietal regions, and mentioned those streamlines may correspond with the temporo-parietal pathways (e.g., TP-SPL) proposed by Kamali et al.^11,23^. The vertical streamlines anterior to VOF that Schurr et al*.* mentioned might also include the IPS-FG as a part of temporo-parietal pathways (Fig. 4H).

The previous tract tracing study showed homologous fiber tracts in the non-human primate, including AF, SLF, ILF, MdLF, and uncinate fasciculus^24^. Although the fusiform gyrus (FG) is considered to be specific to hominoids, the presumptive monkey homologue of human FFA (fusiform face area) and adjacent PPA (parahippocampal place area) are possibly located near the posterior STS (superior temporal sulcus) of the temporal lobe^25^. By injecting tracers to the IPS in rhesus monkeys, Cavada et al. reported the posterior part of the IPS is interconnected with multiple vision‐related areas, including the superior temporal polysensory area (STP) in the upper bank of the STS, the ventral bank of the STS, and the inferotemporal cortex (IT)^26^. By tracing experiments in monkeys, Borra et al. also showed the two main intraparietal areas, AIP and LIP, are preferentially connected with IT areas in the lower bank of the STS, whereas visuomotor IPL convexity areas are preferentially connected with the STP in the upper bank of STS^27^. These results might suggest some connections between the IPS and STS surrounding areas in monkeys could functionally correspond to the IPS-FG in humans.

In the quantitative tractography analysis of IPS-FG, the major streamlines in the left hemisphere interconnect the posterior IPS areas (IP1, IPS1, VIP, MIP) and the FG (FFC, PIT, VVC, TF), while those in the right hemisphere connect the posterior IPS areas (IP1, IPS1, VIP, MIP) and the FG (TF, PHA2, PHA3, FFC, VVC) (Fig. 5C,D). We showed the slightly rightward lateralization of IPS-FG (Fig. 5E). Although there is slightly rightward asymmetry in some connections of the IPS-FG tractography (Fig. 5C,D), we found the bilateral stronger connections between the posterior IPS (i.e., IP1, IPS1) areas and the anterior-to-middle FG (i.e., TF, FFC, VVC, PHA2, PHA3). The FG, including several areas (i.e., TF, FFC, VVC, PHA2-3, VMV2-3, PIT, V8), covers a core network conducting different cognitive tasks (e.g., object recognition, visual language perception, visual attention) with lateralized specific object categories (e.g., words, faces, places, and bodies)^6,7^. In particular, VVC (visual ventral cortex) and FFC (fusiform face complex) cover the region related to the perception of face, body, and word with specific lateralization. The area TF, centered in the anterior FG, could link to motor and language recognition tasks^19^. The area PHA2 and PHA3, in the collateral sulcus, might relate to visuospatial processing and episodic memory^28,29^. Since the right hemisphere is well-known to be dominant in the visuospatial attention^30–32^, the stronger connection of IPS-FG with PHA2 and PAH3 in the right hemisphere could suggest its role in visuospatial attention or memory (Fig. 5C). The Area PIT (posterior inferior temporal), located at the most posterior portion of the fusiform gyrus (Fig. 4A), is linked to the analysis of color and also associated with visual word form area (VWFA) in the left posterior occipitotemporal sulcus^33,34^. Interestingly, we found the stronger connection between the posterior IPS (IP1, IPS1) and the PIT, especially in the left hemisphere (Fig. 5C,D, Supplementary Fig. S6A,B). Previous case reports showed the left lesion in the ventral occipito-temporal cortex, including PIT, resulted in the alexia^35,36^. Collectively, IPS-FG could convey a variety of high-level visual information, including words, to the IPS areas, and play a role in the visual word recognition and visuospatial attention reciprocally.

The posterior parietal cortex (PPC), including the IPS, is considered as a hub for sensorimotor integration. The lesions to the IPS and the SPL in the PPC results in the neurological deficit called Balint’s syndrome (optic ataxia)^2–4^ as described. It establishes a head/body-centered coordinate system through both visual and proprioceptive input with an eye-centered coordinate system^2,37^. IPS is also related to a wide range of cognitive and sensorimotor processes, including attention, working memory, and decision-making^5^. In non-human primates, the anterior IPS region (AIP) is involved in the visuomotor transformation. The lateral IPS region (LIP) is related to eye movement, while the more posterior region (CIP, caudal intraparietal area) is linked to perceptual representations. The IPS-FG appeared to show relatively stronger connectivity in the posterior IPS (i.e., IP1, IPS1) (Fig. 5C,D). Although which of human individual IPS area directly corresponds to the subdivision of monkey IPS (AIP, LIP, CIP) is still controversial^2^, the posterior IPS tends to relate to the retinotopically defined visual regions and superior temporal gyrus, while anterior IPS is linked to prefrontal regions such as functionally defined FEF (frontal eye field) by human neuroimaging studies^2,38^. These results support the functional roles of the posterior IPS are related to the visual cortex in humans.

On the other hand, a fronto-parietal network, including IPS and frontal cortex, is implicated in attentional orienting^39^. The top-down (goal-directed) attentional system might be linked to the dorsal parietal cortex (i.e., SPL) and superior frontal cortex, while bottom-up attentional system is lateralized to the ventral parietal cortex (i.e., IPL) and inferior frontal cortex in the right hemisphere^40^. In fact, a top-down flow of attentional signals from IPS-1 and IPS-2 areas are transmitted to the early visual cortex^41^. Recently Kay and Yeatman found the IPS could be the source of top-down modulation to the VTC possibly thorough the VOF by human neuroimaging studies. They estimated the source is localized in the posterior IPS (IPS-0, IPS-1)^5^. Our dissection (Fig. 3C) and tractography study (Fig. 5C,D) showed the major dorsal projections of IPS-FG could originate from the posterior IPS (e.g., IPS1), with slightly right asymmetry, raising a possible role of IPS-FG in the top-down attentional modulation in parallel with VOF. In addition, the perceptual decision making is a fundamental cognitive ability in which sensory information provides the basis for the selection of one among many alternatives. A network of brain regions was proposed important for such tasks, including sensory areas (posterior cortex), integrative regions (parietal cortex), and response-related regions (frontal lobe)^42,43^. Based on the theory that perceptual decision making is modulated by top-down factors such as attention, Kayser et al. showed the top-down attentional input from the IPS has a significant influence on the visual sensory cortex by human neuroimaging studies^42,43^. As described, the IPS-FG harbors the major streamlines from the posterior IPS (e.g., IP1, IPS1), projecting to category-specific high-level visual areas on the VTC (Fig. 5C). Recently Assem reported that IP1 area is consistently involved in various tasks as a core of fronto-parietal concentrated "multiple-demand (MD)" system^44,45^. Given MD system would function as a common attentional control system across multiple cognitive tasks^44,45^, IPS-FG could be relevant to the top-down attentional signals to modulate the category-specific perceptual decision making in various cognitive tasks. In fact, the human fMRI study showed that feature-specific working memory (WM) representations are encoded by a broadly distributed network of sensory and fronto-parietal cortical areas. The activity in fronto-parietal cortex, including prefrontal cortex and SPL, is elevated during active memory storage, reflecting top-down biasing signals and possibly encoding feature-specific WM^46^. These results suggest the IPS-FG could transmit the top-down biasing signals to encode feature specific visual WM reciprocally.

The present study has limitations. Firstly and importantly, post-mortem dissection suffers from drawbacks such as the highly observer-dependent definition of areal borders and identification of fiber bundles with the lack of functional information as well as interhemispheric interactions. The demonstration of one fiber system often results in the destruction of other fiber systems^47^. It therefore suffers largely the same limitations of tractography (e.g., prone to false positive/negative reconstructions, no cortical terminations, dissector dependent). On the other hand, diffusion-MRI tractography suffers from several drawbacks, including multiple artifacts due to “crossing, branching, merging, and termination” pitfalls^48^.

In summary, we isolated a distinct association fiber tract “IPS-FG” to interconnect the posterior IPS with the FG by white matter dissection. The major dorsal cortical projections arose from the posterior IPS and projected to the FG with slightly rightward asymmetry by tractography. From the anatomical assumption, IPS-FG could play a role in the sensorimotor integration for visually guided actions as well as in the top-down modulation of diverse cognitive functions.

## Material and methods

### White matter dissection

Four normal cerebral hemispheres (two left sides, two right sides) from human cadavers (age range 60–80 years) donated to the Chiba University were studied. White matter dissection was performed according to a modified Klingler’s technique as previously described^8^. After fixed in 10% formalin solution for at least 40 days, brains were washed under running water for several hours to remove the formalin. The pia mater, arachnoid membrane, and vessels of the specimens were carefully removed, and the hemispheres were frozen at − 15 ℃ for 7 days. The specimens were allowed to thaw and then stored in the refrigerator once more for 5 days, as this protocol facilitates white matter dissection. Major anatomical landmarks were identified with needle pins before dissecting the brain. The specimens after thawing were dissected in a stepwise manner from the lateral surface to the medial surface under the magnification loupe (× 3.0) with wooden spatulas.

### NTU-90 brain atlas

NTU-90 brain atlas was constructed by averaging 90 diffusion spectrum imaging (DSI) datasets in the ICBM-152 space, which were provided by the Advanced Biomedical MRI Lab, National Taiwan University Hospital^17,49^.

### The human connectome project (HCP) data

The diffusion dataset in the present study was provided by the Human Connectome Project (https://humanconnectome.org) and WU-Minn Consortium (Principal Investigators: David Van Essen and Kamil Ugurbil; 1U54MH091657). The HCP-1021 template^18^ was averaged from a total of 1,021 subjects’ HCP data from the WU-Minn HCP Consortium (Q1–Q3, 2014) and distributed under the WU-Minn HCP open access data use term. A multi-shell diffusion scheme was used, and the b-values were 1,000, 2,000, 3,000 s/mm^2^. The number of diffusion sampling directions were 90, 90, and 90, respectively. The in-plane resolution was 1.25 mm. The slice thickness was 1.25 mm. The diffusion data were reconstructed in the MNI space using q-space diffeomorphic reconstruction to obtain the spin distribution function^18^.

### Diffusion-MRI tractography

Publicly available imaging data from the Human Connectome Project (https://humanconnectome.org) and NTU-90 brain atlas were analyzed with the DSI Studio software (https://dsi-studio.labsolver.org). 60 healthy unrelated subject data from HCP dataset were analyzed in the present study (Subject IDs: 102614, 103212, 103414, 103818, 104820, 104416, 104012, 105923, 105620, 105115, 105014, 106824, 106521, 107321, 107018, 108323, 109830, 110411, 110007, 112819, 112516, 112314, 111716, 111514,111413, 111312, 111211, 111009, 110613, 163129, 117930, 117324, 117122, 117021, 116,726, 114621, 114419, 119833, 119732, 119126, 119025, 118932, 118831, 118730, 118528, 118225, 118124, 114318, 114116, 113,821, 113619, 113316, 112920, 120010, 304020, 587664, 919966, 952863, 990366, 996782).

The HCP MMP1.0^19^ was originally created in the CIFTI format, which is a surface-based coordinate system (“greyordinates”), therefore it is difficult to perform tractography analysis using ROIs created in the CIFTI format^50^. To convert all 180 areas (HCP MMP1.0) from a surface-based coordinate system to volumetric coordinates, we used the HCP MMP1.0 atlas built in DSI Studio.

Deterministic fiber tracking was conducted as previously described^21,51^. In brief, the reconstruction of tractography was performed with DSI studio (https://dsi-studio.labsolver.org/) by a two ROI (region of interest) approach, in which fiber tracts were generated by whole brain seeding and the tracts ending in ROIs were selected for analysis. The parameters for fiber tracking included a step size of 0.8 mm, a minimum and maximum fiber length of 20 mm and 800 mm respectively, and a turning angle threshold of 75°. When multiple fiber orientations existed in the current progression location, the fiber orientation most congruent with the incoming direction and forming a turning angle smaller than 75° was selected to determine the next moving direction. To smoothen each track, the next moving directional estimate of each voxel was weighted by 50% of the previous incoming direction and 50% of the nearest fiber orientation^51^. This progression was repeated until the quantitative anisotropy (QA) of the fiber orientation dropped below threshold (0.06), until fiber tract continuity no longer met the progression criteria, or until tracking reached to 10,000,000 seeds. For the IPS-FG tractography, the IPS areas (AIP, MIP, VIP, IP1, IP2, IPS1, LIPv, LIPd) and the fusiform gyrus (TF, FFC, VVC, PIT, V8, PHA2, PHA3 VMV2, VMV3) were positioned as the ROI masks of end points. For other fasciculi, including AF and VOF, the automatic fiber tracking function in the DSI Studio were used for reconstructing tractography. For pAF tractography, inferior parietal lobule (AG, SMG) and temporal lobe (ITG, MTG, STG) were served as the ROI masks of end points. For TP-SPL tractography, the parameters in the previous reports^9,11^ were used for fiber tracking. The cortical segmentation of DSI studio’s built-in atlas were visually assessed for accuracy by trained operators. We especially assessed the atlas-based segmentation in DSI studio by checking the IPS areas are localized along the intraparietal sulcus in each brain. We also checked the fusiform gyrus (VVC and FFC) is segmented between CoS (collateral sulcus) and OTS (occipitotemporal sulcus) in each brain.

The fiber orientation distribution function (colored by orientation) in HCP1021 brain template was computed with the default parameters as implemented in MRTrix 3.0^20^.

### Streamline density maps and Group-level integrated tractography

The streamline density of each subject's tractography was obtained by the DSI studio's function using the default parameters. Streamline density maps of the total 60 subjects’ results were registered to a common template space and added together with DSI studio. In regard to the group-level integrated tractography, the individual tractography were converted to binary spatial images (streamline diameter of 0.06 mm), and the stacked images were transformed to the template space^52^.

### Quantitative tractography analysis and connectogram

The quantitative tractography analysis was conducted as previously described^21^. Briefly, the ‘connectivity matrix’ function in DSI studio was used to generate matrices representing the number of fibers ending within regions of a per-subject aligned HCP MMP1.0 atlas. The bilateral connectivity matrices were generated per subject over the 60 individual subjects. The number of fiber tracts corresponding to each respective connection were divided by the total number of fibers per subject. The mean values of 60 subjects were then scaled to obtain a connection index (CI) between 0 and 100, with 0 representing no connectivity, and 100 representing strongest relative connectivity to a particular atlas region. The volumetric connection index (CI) was generated in the same manner using the volume. To measure the volume, the number of voxels occupied by each fiber trajectory (streamlines) were calculated.

Index values of the number and the volume of tracts were then used to generate connectograms by CIRCOS software (https://mkweb.bcgsc.ca/tableviewer/) as described^21,22^. Connectograms provide a unique method of visualizing network topology by demonstrating weighted strength of connectivity between segmented brain regions.

### Laterality index

Laterality index (LI) was calculated in each subject by using the formula: LI = (L − R) / (L + R). L; left, R; right. The LI ranged from − 1 (completely right-lateralized) to + 1 (completely left-lateralized) as previously described^53^.

### Ethics

Informed consent for cadaver use for research and education purposes was acquired from the members of the Whole-Body Donation Registry at Chiba University and their families. The protocol was approved by the Research Ethics Committee of Chiba University School of Medicine.

This study was carried out in accordance with the “Guidelines for Cadaver Dissection in Education and Research of Clinical Medicine” by Japan Surgical Society and Japanese Association of Anatomists.

## Supplementary information

Supplementary Information
