# Supplementary material for: Identification of a distinct association fiber tract “IPS-FG” to connect the intraparietal sulcus areas and fusiform gyrus by white matter dissection and tractography
Source: Sci Rep. 2020 Sep 23;10:15475. doi: 10.1038/s41598-020-72471-z (PMC7511306; doi:10.1038/s41598-020-72471-z)

Identification of a distinct association fiber tract "IPS-FG" to connect the intraparietal sulcus areas and fusiform gyrus by white matter dissection and tractography

Tatsuya Jitsuishi, Atsushi Yamaguchi *

Materials and methods

Probabilistic tractography

For the probabilistic tractography, we used MRtrix3 software package (http://www.mrtrix.org)(ref.#1) based on the protocol for using HCP datasets (https://mrtrix.readthedocs.io/en/0.3.16/tutorials/hcp_connectome.html). One representative subject (ID I04012) was used. We first generated a tissue-segmented image appropriate for ACT (Anatomically-Constrained Tractography) (ref. #2). We next estimated the response functions from the preprocessed diffusion-weighted images to estimate fiber orientation distributions (FOD) based on the constrained spherical deconvolution (CSD) using iFOD2 algorithm (dwi2fod). Then we generated tractography using "tckgen" in the command line (seed = whole brain, default step size, maximum harmonics order = 8, termination criteria: exit the brain or when the CSD fiber-orientation distribution amplitude was < 0.06, maximum number of fibers=50M), followed by SIFT (Spherical-deconvolution Informed Filtering of Tractograms) to improve the quantitative nature of whole-brain streamlines reconstructions.

If the streamlines did not end in 'inclusion ROIs' (IPS areas, FG) or enter 'exclusion regions' (AF, VOF, Corpus Callosum (CC)), they were deleted using "tckedit" in MRtrix3 command [$ tckedit 50M.tck (input_file) (output_file) -include IPS –include FG –exclude AF –exclude VOF –exclude CC]. The 'inclusion ROIs' (IPS areas, FG) and 'exclusion regions' (AF, VOF, CC), attained by DSI studio, were mapped to the diffusion space of subject as described (ref. #3, #4). An 'exclusion region' of the corpus callosum (CC) was manually drawn on the mid-sagittal slice of the FA image (ref. #3). These regions were carefully reviewed to ensure the accuracy of the anatomic registration and parcelation by "mrview" of MRtrix3.0.

Reference.

#1. Tournier, J.-D. et al. MRtrix3: A fast, flexible and open software framework for medical image processing and visualisation. Neuroimage 202, 116137 (2019).

#2. Smith, R. E., Tournier, J.-D., Calamante, F. & Connelly, A. Anatomically-constrained tractography: improved diffusion MRI streamlines tractography through effective use of anatomical information. Neuroimage 62, 1924–1938 (2012).

#3. Zhang, H., Bao, Y., Feng, Y., Hu, H. & Wang, Y. Evidence for Reciprocal Structural Network Interactions Between Bilateral Crus Lobes and Broca's Complex. Front Neuroanat 14, 27 (2020).

#4. Fekonja, L. et al. Manual for clinical language tractography. Acta Neurochir (Wien) 161, 1125–1137 (2019).

Supplementary Figure S1

White matter dissection of IPS-FG in the right hemisphere.

(A) The lateral view of right hemisphere after removal of the meninges and vessels, with representative anatomical landmarks. (B) The lateral view after dissection to expose the fiber bundles of IPS-FG. The frontoparietal and temporal opercula around the insula were removed. (C) The magnified images of the posterolateral corner of the brain. (D) Isolation of IPS-FG in the right hemisphere. Fiber bundles of ILF/IFOF were exposed after removal of IPS-FG. (E) Ventral temporal cortex (VTC) of the brain, showing FG and CoS. "V3A/B, IPS-0, IPS-1, and IPS-2 area" are the anatomy-based possible visual map. AF; arcuate fasciculus, FG; fusiform gyrus, VOF; vertical occipital fasciculus, ILF; inferior longitudinal fasciculus, IFOF; inferior fronto-occipital fasciculus, IPS; intraparietal sulcus, POS; parieto-occipital sulcus, TOS; transverse occipital sulcus. A; anterior, P; posterior, S; superior.

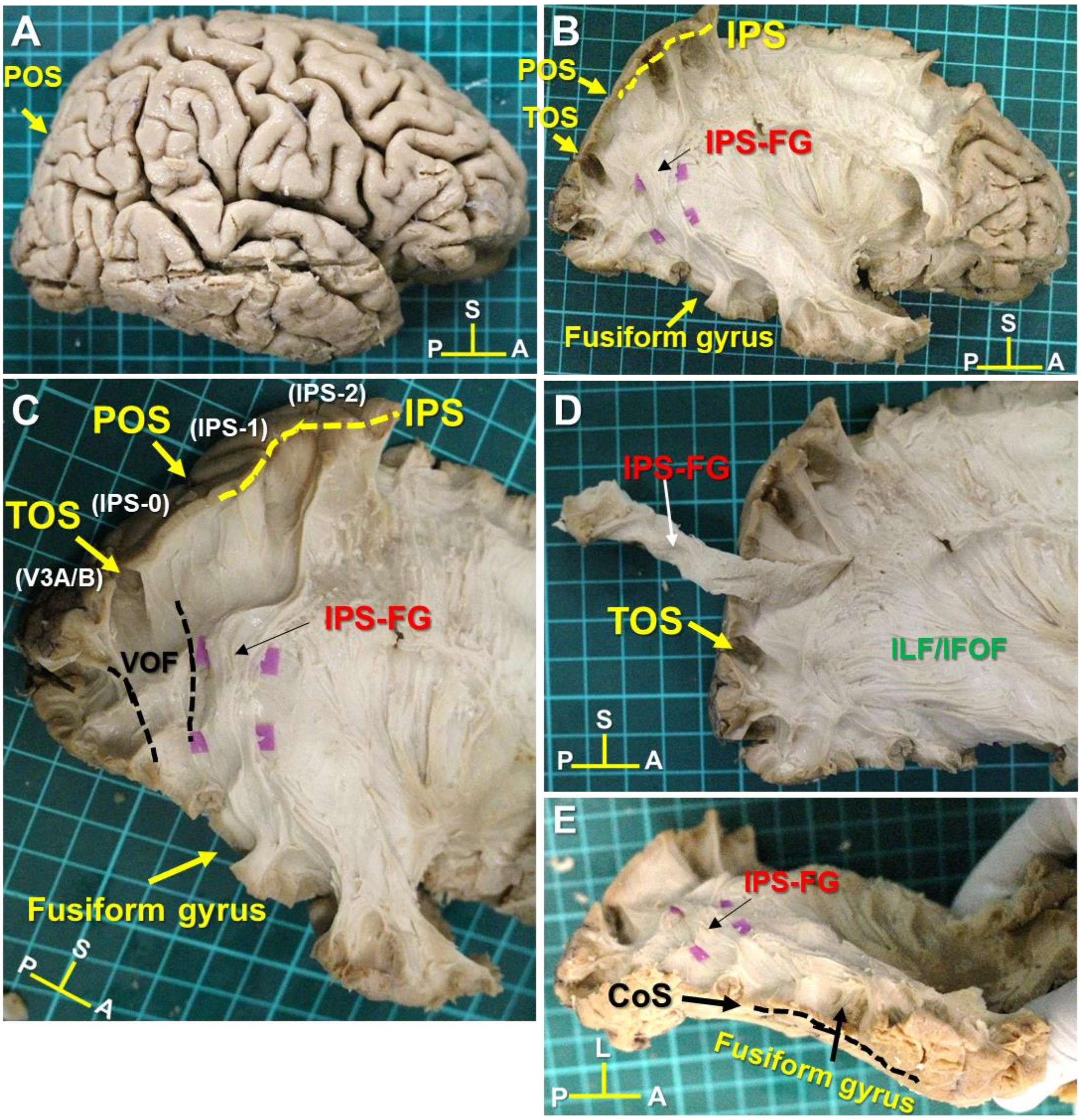

Supplementary Figure S2

White matter dissection of IPS-FG in the right hemisphere.

(A) The lateral view of right hemisphere after removal of the meninges and vessels, with representative anatomical landmarks. (B) The lateral view after dissection to expose the fiber bundles of IPS-FG and VOF. (C) The magnified image of the posterolateral corner of the brain. (D) Ventral temporal cortex (VTC) of the brain, showing FG and CoS.

"V3A/B, IPS-0, and IPS-1 area" are based on the anatomy-based possible visual map.

AF; arcuate fasciculus, FG; fusiform gyrus, VOF; vertical occipital fasciculus, ILF; inferior longitudinal fasciculus, IFOF; inferior fronto-occipital fasciculus, IPS; intraparietal sulcus, POS; parieto-occipital sulcus, TOS; transverse occipital sulcus. A; anterior, P; posterior, S; superior, L; lateral.

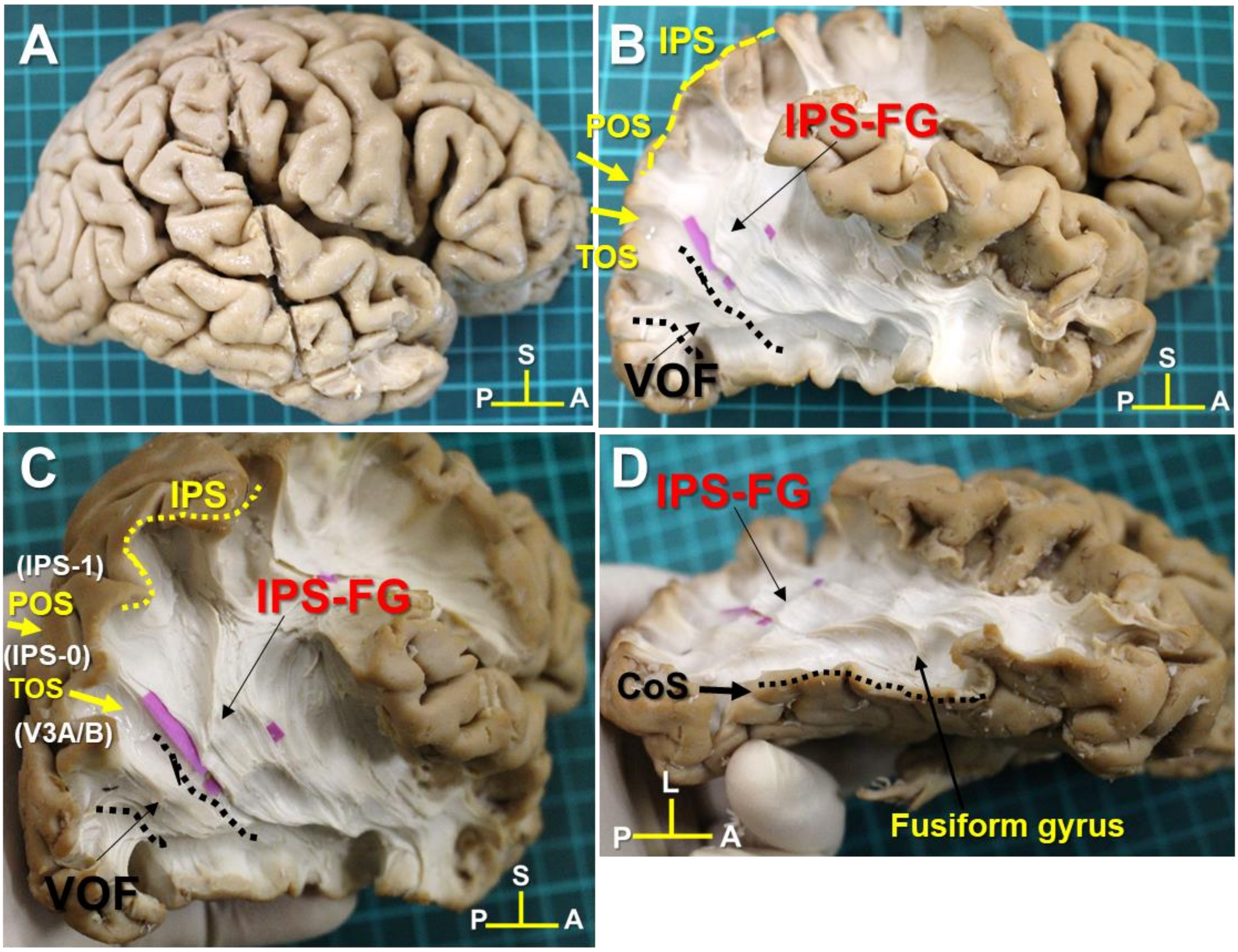

Supplementary Figure S3

White matter dissection of IPS-FG in the left hemisphere.

(A) The lateral view of left hemisphere after removal of the meninges and vessels, with representative anatomical landmarks. (B) The lateral view after dissection to expose the fiber bundles of IPS-FG and AF. The frontoparietal and temporal opercula around the insula were removed. (C)(D) Caudal view to show the ISP-FG to fall in the medial bank of IPS. "V3A/B, IPS-0, IPS-1, and IPS-2 are" are based on the anatomy-based possible visual map.

AF; arcuate fasciculus, FG; fusiform gyrus, VOF; vertical occipital fasciculus, ILF; inferior longitudinal fasciculus, IPS; intraparietal sulcus, POS; parieto-occipital sulcus, TOS; transverse occipital sulcus. A; anterior, P; posterior, S; superior.

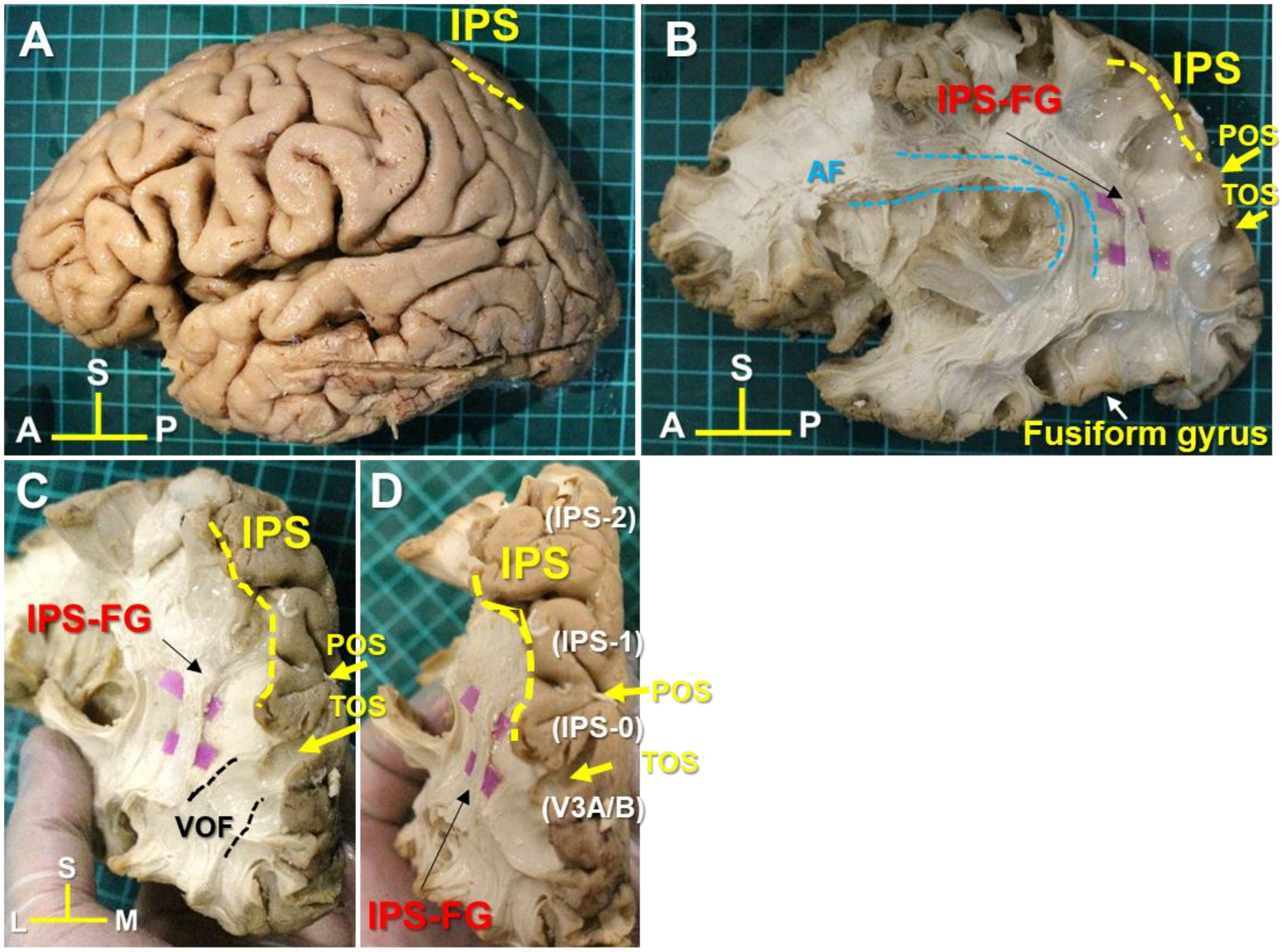

Supplementary Figure S4

Representative images of individual IPS-FG tractography.

Five subjects' images of IPS-FG tractography were shown (left lateral, right lateral, coronal, and axial view).

Subject IDs (#1,1106521; #2,117021; #3, 117122; #4, 112516, #5, 118932).

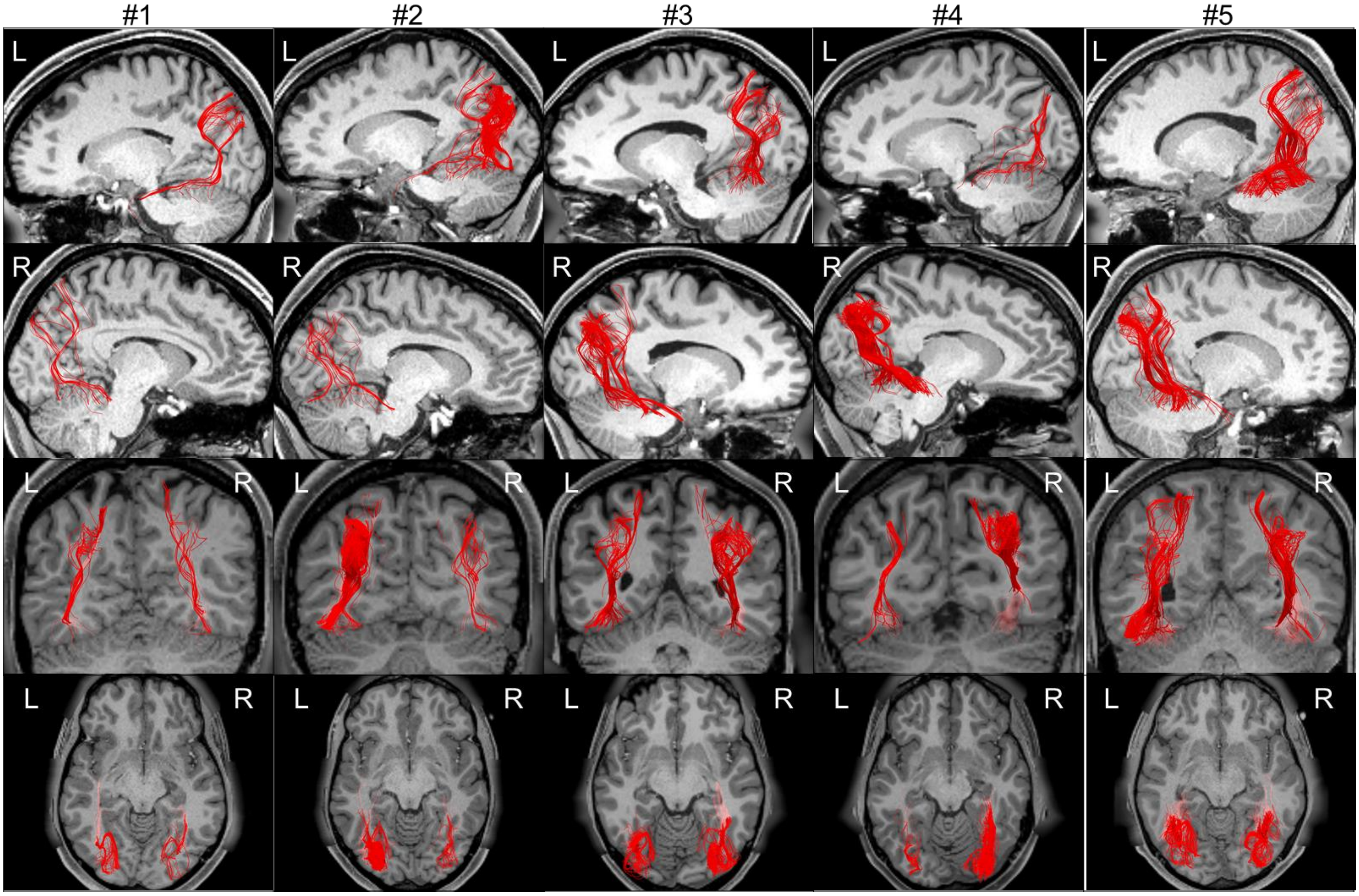

Supplementary Figure S5

Comparison between probabilistic (MRtrix3.0) and deterministic (DSI studio) tractography of IPS-FG.

(A)Visualizations for ROI placements to reconstruct IPS-FG tractography by MRtrix3.0. The 'inclusion ROIs' (IPS areas, FG) were overlaid on the T1-weighted image. (B) Visualizations for 'inclusion ROIs' (IPS areas, FG) and 'exclusion regions' (AF, VOF) with IPS-FG tractography, overlaid on the T1-weighted image.

(C)(D) Visualizations for IPS-FG tractography by MRtrix3.0. The main structures (opacity=0.2) of coronal and lateral view were overlaid on the T1-weighted image, respectively.

(E)(F) Visualizations for IPS-FG tractography by DSI studio, to compare the tractography between MRtrix3.0 (probabilistic algorithm) (C)(D) and DSI studio (deterministic algorithm) software tool.

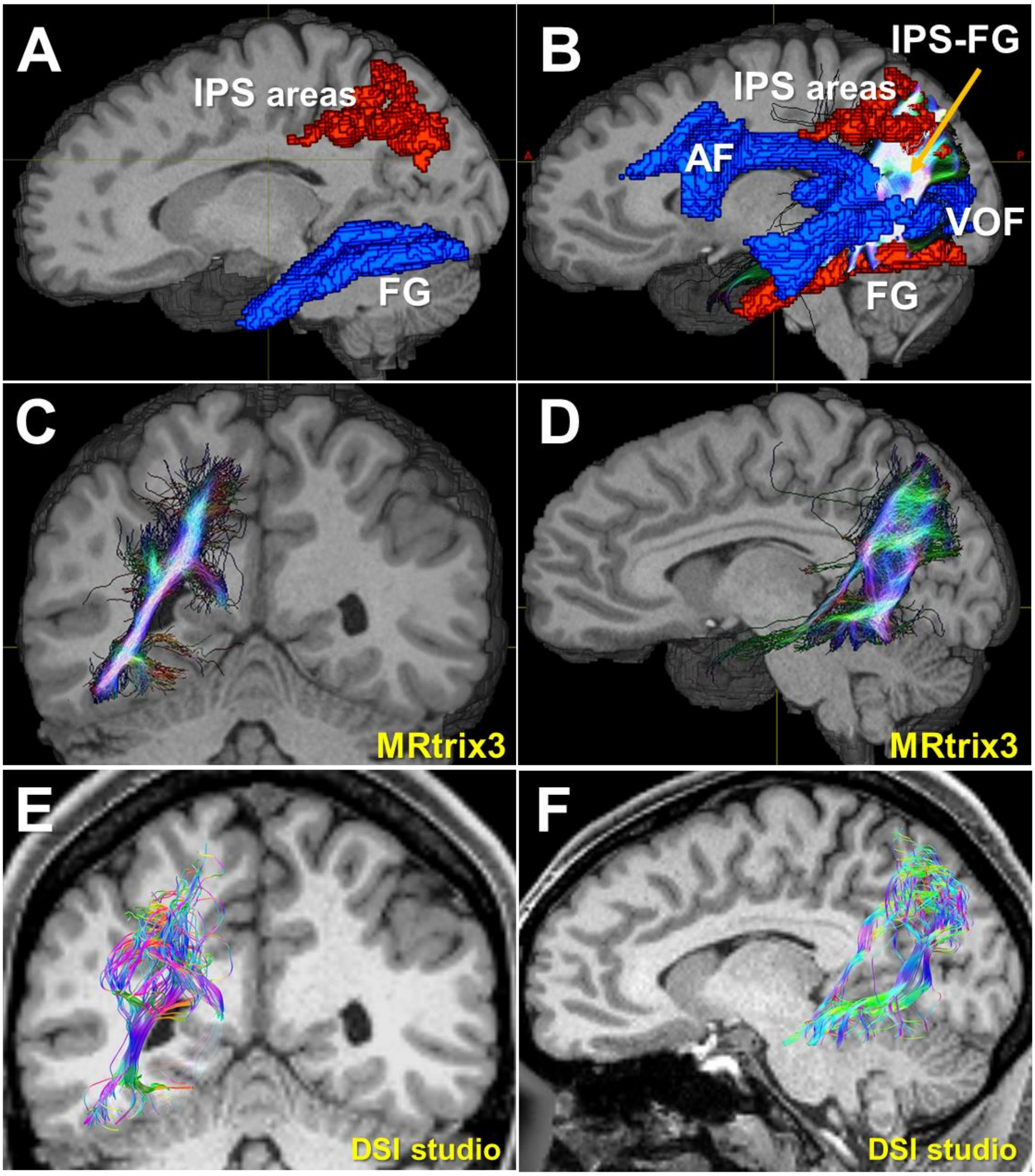

Supplementary Figure S6

Connection Index (the volume of tracts), Connectogram, Laterality Index (LI).

(A) The tables indicate the average Connection Index (CI) of the tract volume for each connection to link IPS areas and FG. Left; left hemisphere. Right; right hemisphere. Darker colors indicate the strength of connection. The data is average of 60 subjects from HCP dataset.

(B) Connectogram representing the bilateral connectivity patterns of IPS-FG, reflecting the tract volume of each connection. Circular color map represents segmented brain regions that each connection interconnects. The arc and ribbon size represent the computed degrees of connectivity (the volume of tracts) between segmented brain regions. The data is average of 60 subjects from HCP dataset. AIP, anterior intraparietal; VIP, ventral intraparietal; MIP, medial intraparietal; IP1, intraparietal 1; IP2, intraparietal 2; IPS1, intraparietal sulcus 1;LIPv, lateral intraparietal ventral, ;LIPd, lateral intraparietal dorsal; PHA2, 3, parahippocampal area 2, 3; V8, visual area 8; PIT, posterior inferior temporal; FFC, fusiform face complex; VVC, ventral visual complex; VMV2, 3, ventro-medial visual areas 2, 3.

(C) Graph indicates the laterality index (LI) for the volume of tracts in each subject (#1-60). The red lowermost lane is the average value of 60 subjects. The *laterality index* (left−right)/(left+right) shows the cerebral asymmetry.

A Connection Index (CI)

Rows: FG, Anterior ↔ Posterior. Columns: IPS areas, Anterior ↔ Posterior.

Left

	AIP	IP2	LIPv	LIPd	VIP	MIP	IP1	IPS1
TF	23.8	13.3	28.7	9.7	39.1	36.8	73.7	43.8
FFC	32.6	25.5	48.1	20.0	75.8	42.9	78.9	56.6
VVC	19.4	16.1	22.5	11.4	56.0	30.8	69.8	49.6
PHA3	15.0	6.9	15.6	2.7	40.7	20.4	55.8	46.0
PHA2	2.2	2.5	3.5	2.2	12.5	9.5	19.6	17.5
VMV3	2.1	0.0	2.9	1.6	14.1	5.4	20.0	11.9
VMV2	0.0	0.0	0.0	1.6	14.6	3.8	8.3	11.2
V8	6.2	2.4	15.5	5.5	38.1	17.3	36.4	34.3
PIT	6.2	6.3	7.2	8.8	34.3	20.6	34.8	32.5

Right

	AIP	IP2	LIPv	LIPd	VIP	MIP	IP1	IPS1
TF	27.5	8.4	34.0	21.7	69.0	77.1	100.0	78.6
FFC	11.6	8.7	24.6	8.4	67.7	46.7	86.1	62.6
VVC	16.2	4.4	19.4	10.1	53.9	49.4	77.4	59.6
PHA3	10.9	2.2	25.2	6.1	30.1	51.0	72.6	52.6
PHA2	6.4	10.8	17.5	8.2	31.6	32.9	74.9	48.5
VMV3	1.6	0.0	2.1	0.0	10.9	2.1	20.6	11.3
VMV2	0.0	1.7	2.6	4.0	11.3	11.7	15.7	21.0
V8	0.0	0.0	0.0	2.1	6.3	6.0	20.4	16.1
PIT	0.0	0.0	2.1	4.0	7.2	13.9	28.2	15.4

B

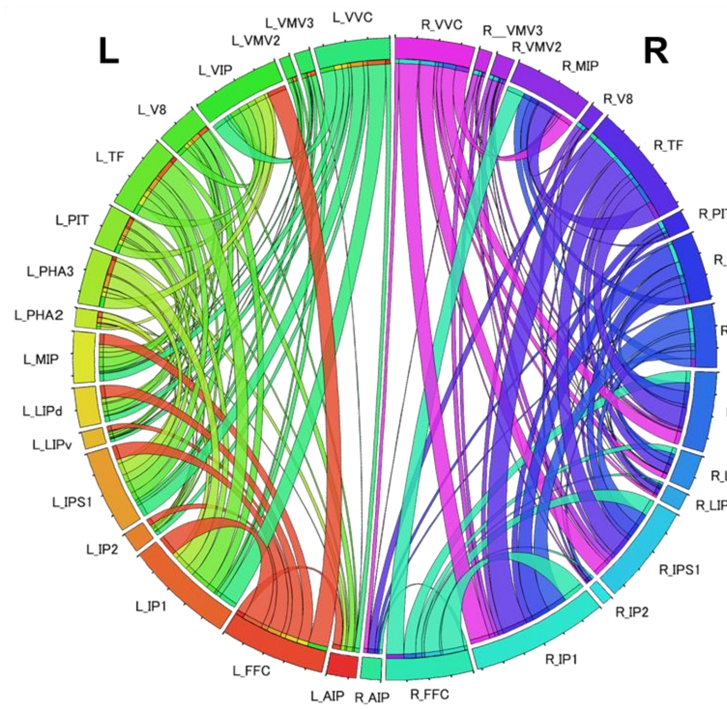

C

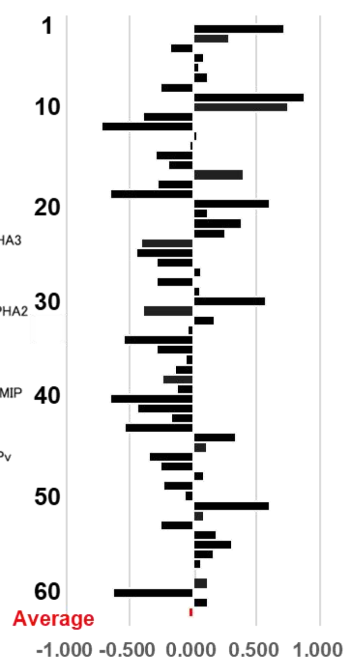

Supplementary Figure S7

Diffusion-MRI tractography to show the anatomical relationship between IPS-FG and TP-SPL in NTU-90 brain atlas.

(A) (B) Left and Right lateral view of IPS-FG (red) with TP-SPL (yellow) tractography overlaid on T1-weighted image, respectively.

(C) (D) Axial and coronal view of IPS-FG (red) with TP-SPL (yellow) tractography, respectively.

The parameters to reconstruct TP-SPL tractography were described previously (Brain Res. 1646:152–159 (2016)).

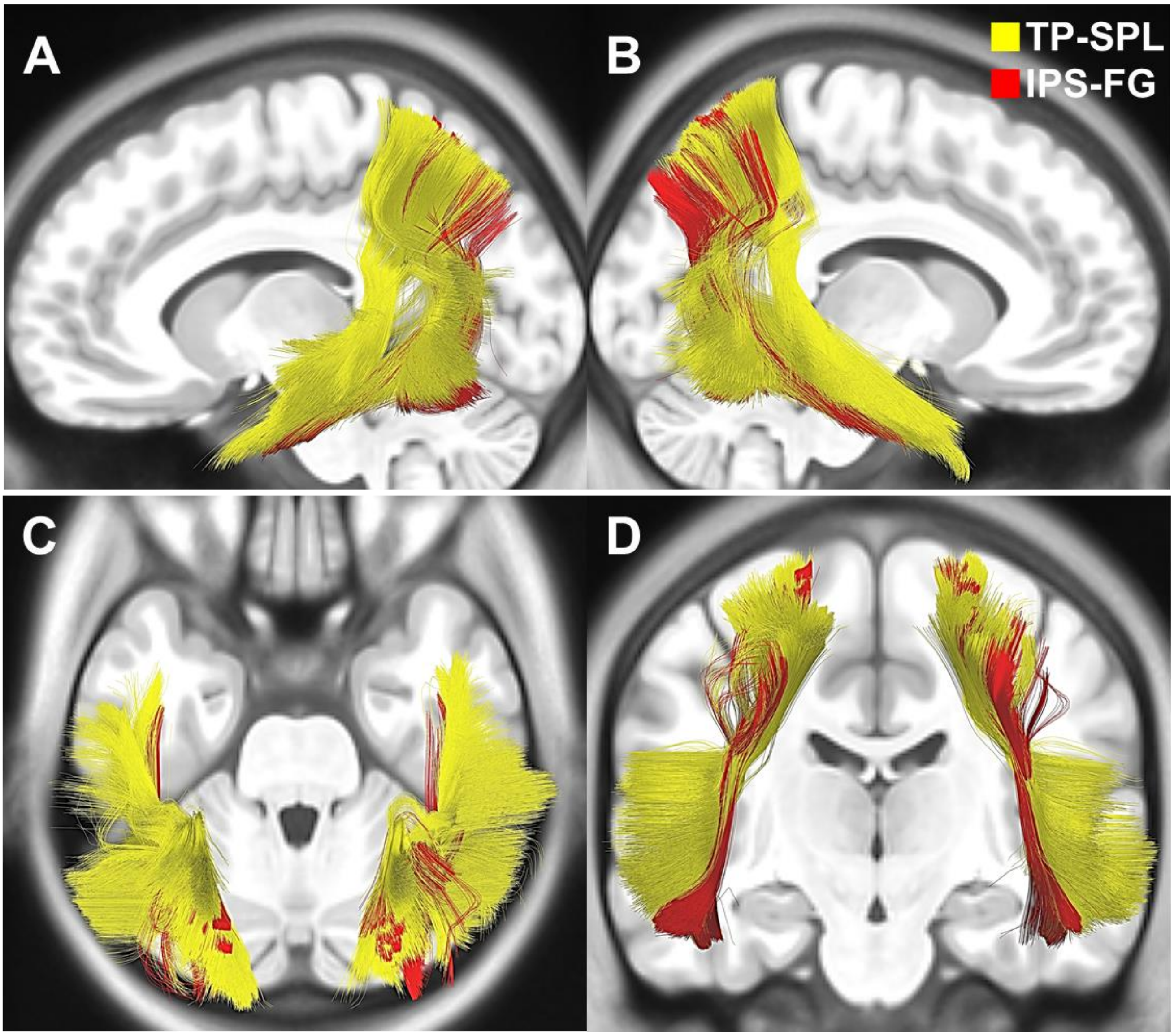

Supplement: Supplementary file 1 — Supplementary Information [file 41598_2020_72471_MOESM1_ESM.pdf]
